# Association between air pollution and cardiovascular mortality in China: a systematic review and meta-analysis

**DOI:** 10.18632/oncotarget.20090

**Published:** 2017-08-09

**Authors:** Lei Zhao, Heng-Rui Liang, Feng-Ying Chen, Zi Chen, Wei-Jie Guan, Jian-Hua Li

**Affiliations:** ^1^ Key Laboratory of Protein Modification and Degradation, School of Basic Medical Sciences, Affiliated Cancer Hospital and Institute of Guangzhou Medical University, Guangzhou, 511436, China; ^2^ The Sixth Affiliated Hospital, Guangzhou Medical University, Guangzhou, 511436, China; ^3^ Nan Shan School, Guangzhou Medical University, Guangzhou, 511436, China; ^4^ State Key Laboratory of Respiratory Disease, National Clinical Research Center for Respiratory Disease, Guangzhou Institute of Respiratory Disease, The First Affiliated Hospital of Guangzhou Medical University, Guangzhou, 510120, China; ^5^ Huashan Hospital, Fudan University, Shanghai, 200040, China; ^6^ QuintilesIMS Asia Medical Oncology, Shanghai, 200032, China

**Keywords:** air pollution, cardiovascular, mortality, China, meta-analysis

## Abstract

Air pollutant levels in many Chinese cities remained significantly higher than the upper limits stated in World Health Organization guidelines. In light of limited evidence in China, we conducted a meta-analysis summarizing the association between acute exposure of air pollution and cardiovascular mortality. We searched PubMed, and CNKI databases etc. for literature published in English or Chinese up to January 2017. Outcomes were pooled and compared using random-effects model. Excess risks (ERs) per 10 μg/m^3^ increase in PM_2.5_, PM_10_, NO_2_, SO_2_ and O_3_ were evaluated. Subgroup analysis was conducted according to lag patterns (lags 0, 1, 2, 0–1, 0–2 days), gender (male vs. female), temperature (cool vs. warm) and age (< 65 vs. ≥ 65). Study bias was detected using Begg’s and Egger’s test. Of 299 articles identified, 30 met inclusion criteria. Each 10 μg/m^3^ increase in the concentration was associated with a higher incidence of cardiovascular mortality for PM_2.5_ (0.68%, 95% CI: 0.39–0.97%), PM_10_ (0.39%, 95% CI: 0.26–0.53%), NO_2_ (1.12%, 95% CI: 0.76–1.48%), SO_2_ (0.75%, 95% CI: 0.42–1.09%), and O_3_ (0.62%, 95% CI: 0.33–0.92%), respectively. Air pollution conferred greater adverse impacts on cardiovascular mortality for longer duration of exposures. Strongest associations were seen for lag 0–1 day of exposure among all pollutants. Female, lower temperature, and age > 65 years were associated with greater risks of cardiovascular mortality for all pollutants. Higher concentrations of air pollutants correlated with a greater short-term increase in cardiovascular mortality. Further high-quality studies in China are urgently warranted to determine the susceptible population, which would offer reference for policy-making to minimize adverse health effects.

## INTRODUCTION

Despite considerable improvement in prevention and management, cardiovascular diseases remain the leading cause of death worldwide. Decomposition of global and regional life expectancy showed the prominent role of reductions in age-standardized death rates for cardiovascular diseases worldwide [1]. According to World Health Organization (WHO), about 17.3 million people died of cardiovascular diseases annually, accounting for > 30% of all-cause mortality [2]. Cardiovascular mortality is the top cause for mortality worldwide, with 3.7 million people aged below 60 years [3]. Factors that trigger cardiovascular events, particularly in susceptible population, represent a major public health concern.

Air pollution is a leading environmental health issue [4]. Higher levels of gaseous components [including nitrogen dioxide (NO_2_), sulfur dioxide (SO_2_), and ozone (O_3_)] and particulate matter with diameter less than 10μm (PM_10_) and 2.5 μm (PM_2.5_) have been associated with poorer cardiovascular health [5, 6]. Short-term exposure to air pollutants led to increased cardiovascular events [7–10]. According to *2002 World Health Report*, approximately 800,000 premature deaths were attributable to air pollution worldwide annually [11]. Moreover, short-term exposures to air pollution have been associated with increased cardiovascular mortality and hospital admissions [7, 12]. China is the major developing country. Air pollutant levels in many cities remain significantly higher than the upper limits stated in the WHO guidelines [13]. Daily concentrations of PM_2.5_ in mega-cities, such as Beijing and Shanghai, may peak to 100–300 μg/m^3^ [14, 15], which far exceeded the upper limit (10 μg/m^3^) endorsed by WHO Air Quality Guideline (AQG) [13].

However, most evidence of the association between short-term effect of air pollution and cardiovascular mortality were derived from North American or West European countries where pollutant levels were much lower [16–18]. Studies conducted in China are very scarce. Importantly, previous findings might not be directly extrapolated to China [19], because adverse effects of air pollution vary with regions and time [20]. Therefore, we conducted a systematic review and meta-analysis summarizing the association between short-term exposure of air pollution and cardiovascular mortality in China.

## RESULTS

### Study selection and identified papers

**Figure 1:**
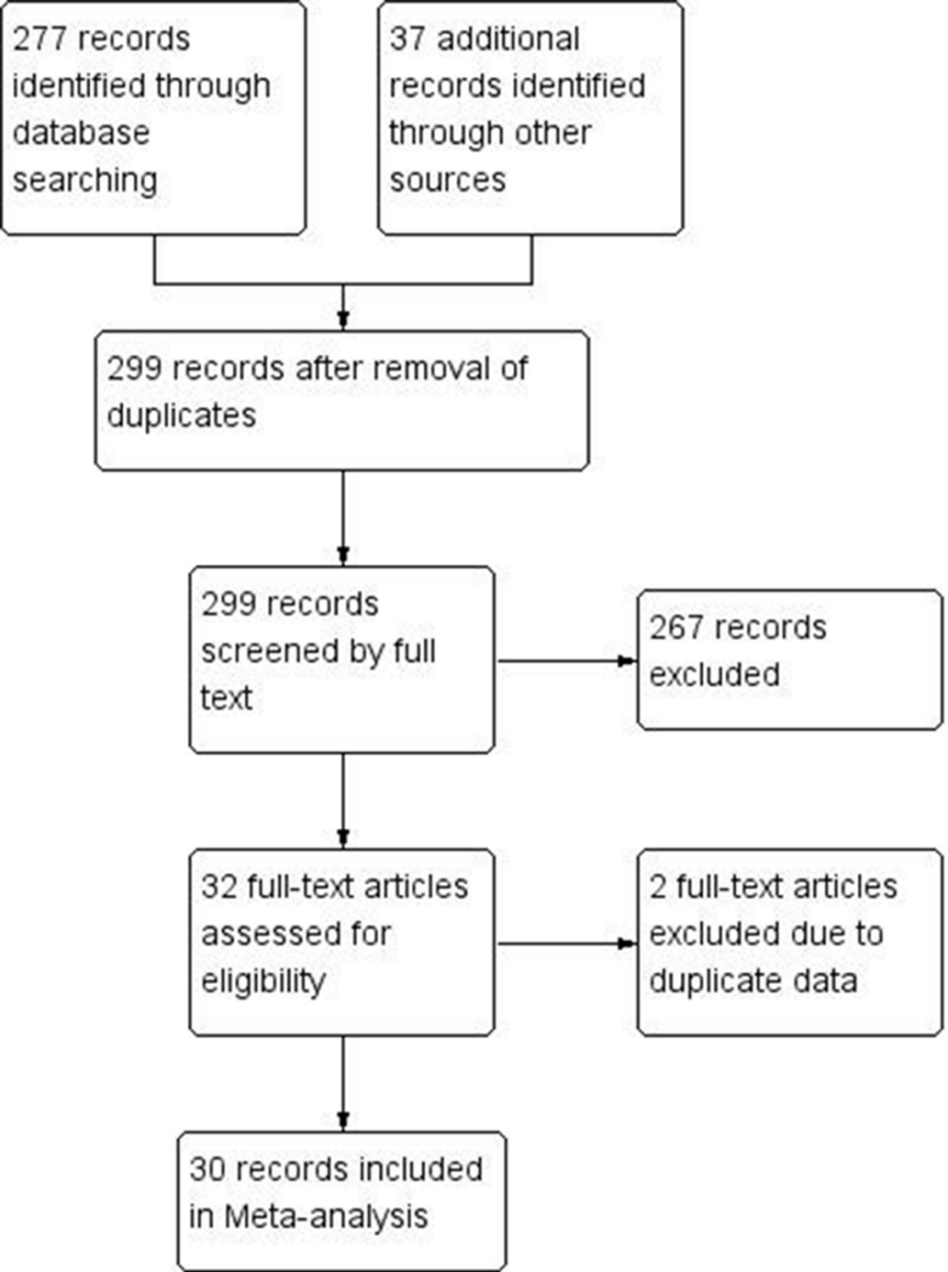
Flow chart of literature search and criterion for inclusion and exclusion of studies

299 records were identified after excluding duplicates. With in-depth review, 30 full-text articles fulfilled the inclusion criteria and were included in the meta-analysis. Details of selection process are presented in Figure 1. The median concentrations of each pollutant was 54 μg/m^2^, 85.3 μg/m^2^, 46 μg/m^2^, 57 μg/m^2^ and 57.88 μg/m^2^ for PM_2.5_, PM_10_, SO_2_, NO_2_ and O_3,_ respectively_._

Across the span of 1998 to 2015, two studies adopted case-crossover design and the remaining were time-series studies. Cardiovascular mortality effects were assessed in association with acute exposure to five major air pollutants, including PM_10_, PM_2.5_, SO_2_, NO_2_ and O_3_. Of these, 17 articles focused on PM_10_, 10 studies were pertinent to PM_2.5_, 12 studies included NO_2_, 9 studies was related to SO_2_ and 6 studies referred to O_3_. Eleven cities were included in our study, which were mainly comprised of provincial capitals in China. Table 1 summarizes the details of studies included in our study. See Supplementary Table 1 and Supplementary Figures 1– 11.

**Table 1:** Contextual details of studies included in the meta-analysis

Study	Year	City	Study period	Study design	Air pollutant	Lags exposure	Model
Xu et al.	2017	Beijing, China	2013	Time-series	PM_2.5_, NO_2_, SO_2_, O_3_, CO	0, 1, 2, 3, 4, 0–1, 0–3, 0–5	GAM
Qin et al.	2016	Zhengzhou, China	2013–2015	Time-series	O_3_	0–1	GAM
Lin et al.	2016	China	2013–2015	Time-series	PM_2.5_, SO_2_, NO_2_, O_3_	0–3	GAM
Xie et al.	2015	Beijing, China	2010–2012	Time-series	PM^2.5^	0, 1, 2, 3, 4, 0–2, 0–4	GAM
Li et al.	2015	Beijing, China	2005–2009	Time-series	PM_2.5_	1,2	GAM
Zhang et al.	2014	Guangzhou, China	2008–2011	Time-series	PM_10_, SO_2_, NO_2_	0–5	GAM
Tong et al.	2014	Tianjin, China	2008–2011	Time-series	PM_10_, SO_2_, NO_2_	0, 0–1	GLM
Yu et al.	2013	Hong Kong, China	1998–2007	Time-series	PM_10_, NO_2_	0–3	GAM
Wang et al.	2013	Tianjin, China	2006–2010	Time-series	PM_10_, SO_2_, NO_2_	0, 1, 2, 0–3	GAM
Huang et al.	2013	Hong Kong, China	1998–2007	Time-series	PM_10_, NO_2_, O_3_, SO_2_	0, 1, 0–3	GAM
Geng et al.	2013	Shanghai, China.	2007–2008	Time-series	PM_2.5_	3	NG
Yu et al.	2012	Guangzhou, China	2006–2009	Time-series	PM_10_, SO_2_, NO_2_	0–1	GAM
Yang et al.	2012	Suzhou, China	2006–2008	Time-series	O_3_	0–1	GAM
Yang et al.	2012	Guangzhou, China	2007–2008	Case-crossover	PM_2.5_	0–1	Logistic
Tao et al.	2012	China	2006–2008	Time-series	PM_10_, NO_2_, O_3_	0–2	GLM
Xia et al.	2012	China	2001–2008	Time-series	PM_10_	0–1	GAM
Huang et al.	2012	Xi’an, China	2004–2008	Time-series	PM_2.5_	0–2	GAM
Chen et al.	2012	China	2001–2008	Time-series	NO_2_	0–1	GLM
Chen et al.	2012	China	2006–2009	Time-series	PM_10_	0–1	GAM
Ma et al.	2011	Shenyang, China	2006–2008	Case-crossover	PM_2.5_	0–1	GAM
Chen et al.	2011	China	2006–2008	Time-series	PM_2.5_, PM_10_	0, 1, 2, 0–1, 0–2	GLM
Qian et al.	2010	Xian, China	2004–2008	Time-series	PM_2.5_	0–1	GAM
Chen et al.	2010	Anshan, China	2005–2007	Time-series	PM_10_, SO_2_, NO_2_	0, 6, 0–1, 0–6	GAM
Chen et al.	2010	Shanghai, China.	2005–2007	Time-series	PM_10_, SO_2_, NO_2_	0, 6, 0–1, 0–6	GLM
Huang et al.	2009	Shanghai, China.	2004–2005	Time-series	PM_2.5_	0	GAM
Cao et al.	2009	Shanghai, China.	2005–2007	Time-series	PM_10_, SO_2_	0, 6, 0–1, 0–6	GLM
Wong et al.	2008	China	2001–2004	Time-series	PM_10_, SO_2_, NO_2_, O_3_	0–1	GLM
Chen et al.	2008	Shanghai, China.	2001–2004	Time-series	PM_10_, SO_2_, NO^2^	0–1	GAM
Qian et al.	2007	Wuhan, China	2001–2004	Time-series	PM_10_, O_3_	0, 4, 0–1, 0–4	GAM
Kan et al.	2007	Shanghai, China	2004–2005	Time-series	PM_2.5_, PM_10_	0–1	GAM

GAM, Generalized additive model. GLM, Generalized linear model. Logistic, Logistic regression model.

### Particulate matter and cardiovascular mortality

**Figure 2:**
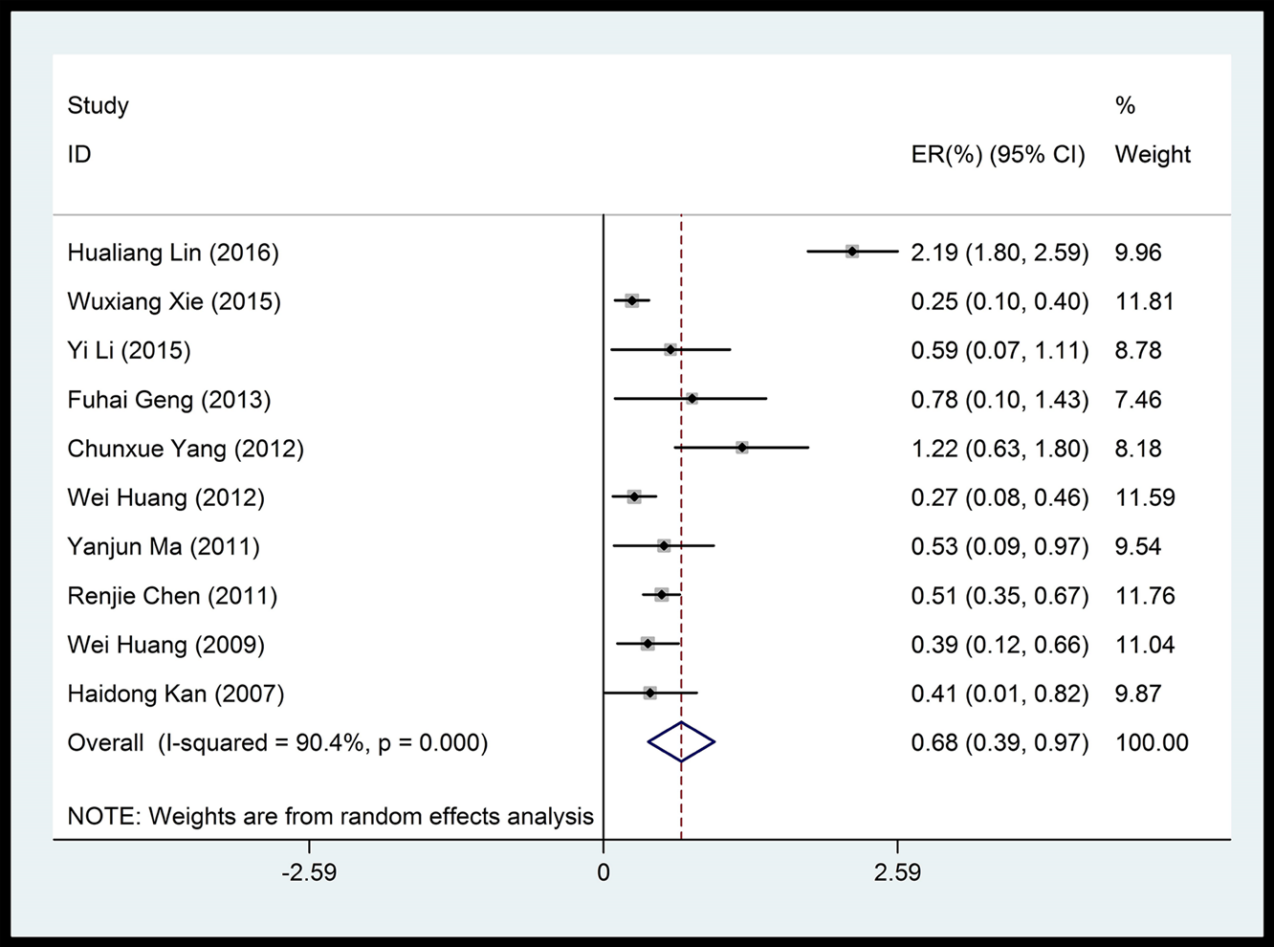
Forest plot of the association between PM_2.5_ and cardiovascular mortality

**Figure 3:**
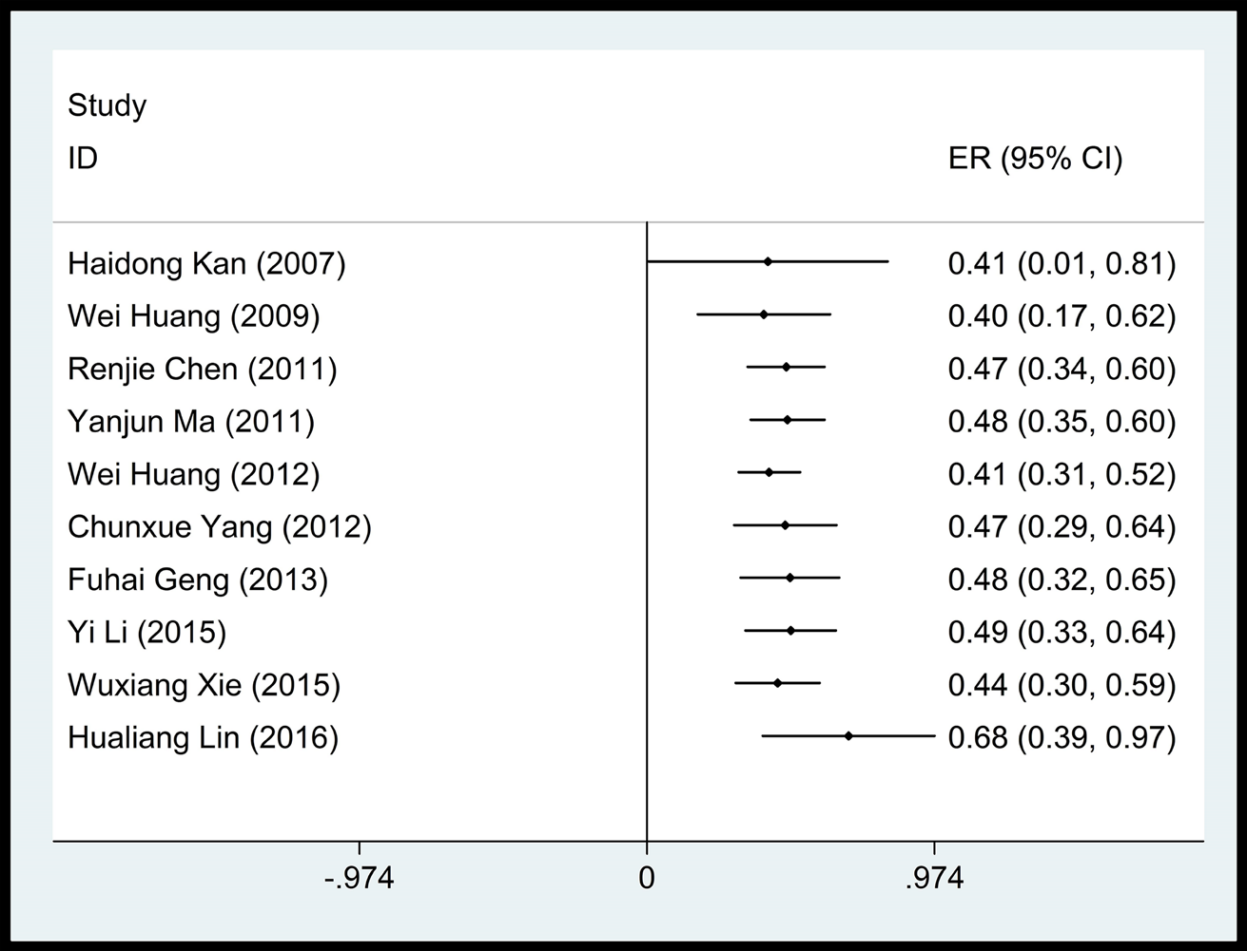
Accumulative meta-analysis of the association between PM_2.5_ and cardiovascular mortality

Overall, we noted significant positive associations between cardiovascular mortality and particulate matters (PM_10_, PM_2.5_). PM_10_, which was the most frequently reported air pollutant, showed a mean of 0.39% (95% CI: 0.26%–0.53%) increase in cardiovascular mortality risk per 10 μg/m^3^ increase in concentration, with a significant heterogeneity among studies (83.6%) (Figure 2). Pooled analysis suggested that the adverse impact of PM_10_ on cardiovascular mortality was independent of the duration of exposure (Figure 3).

**Figure 4:**
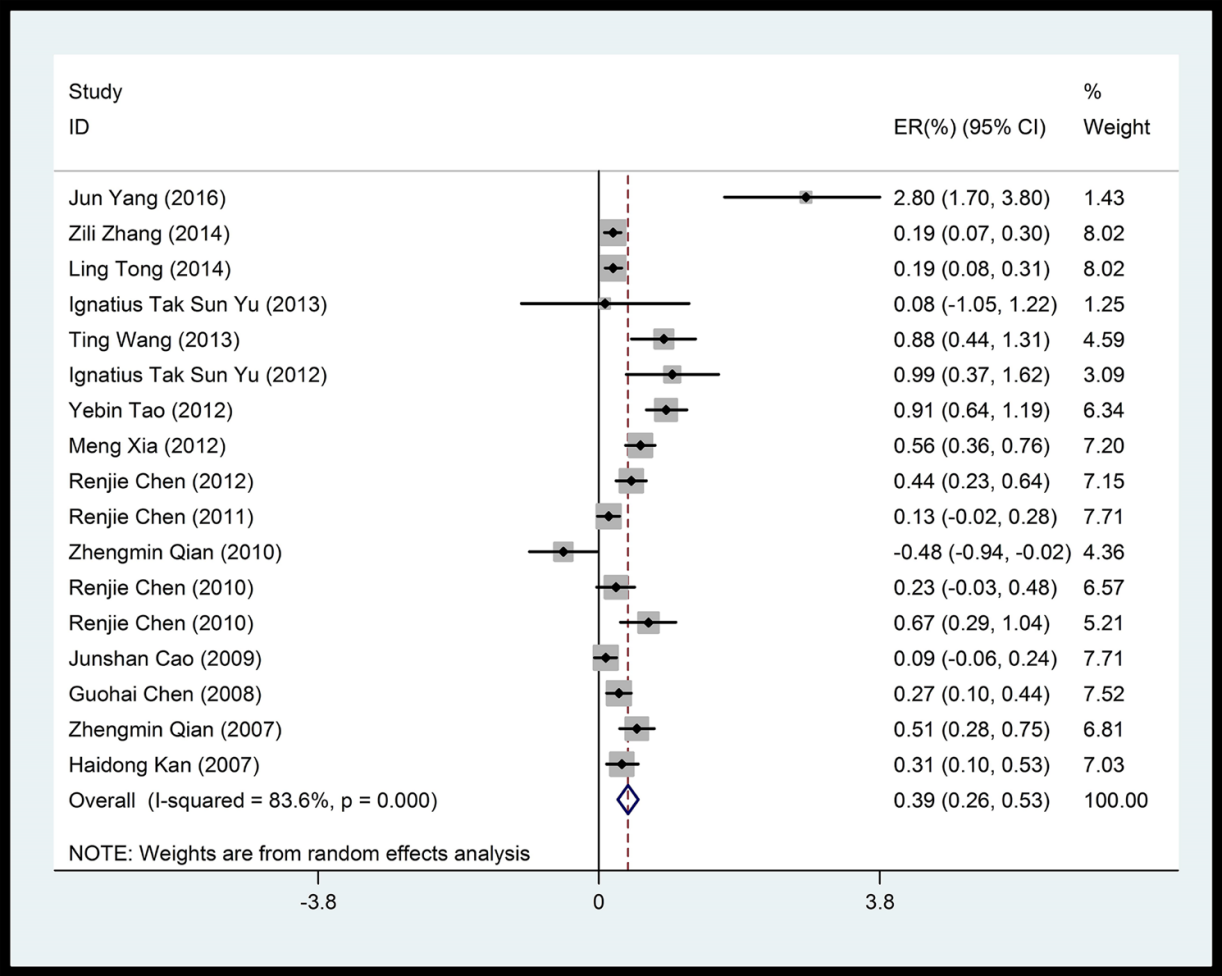
Forest plot of the association between PM_10_ and cardiovascular mortality

**Figure 5:**
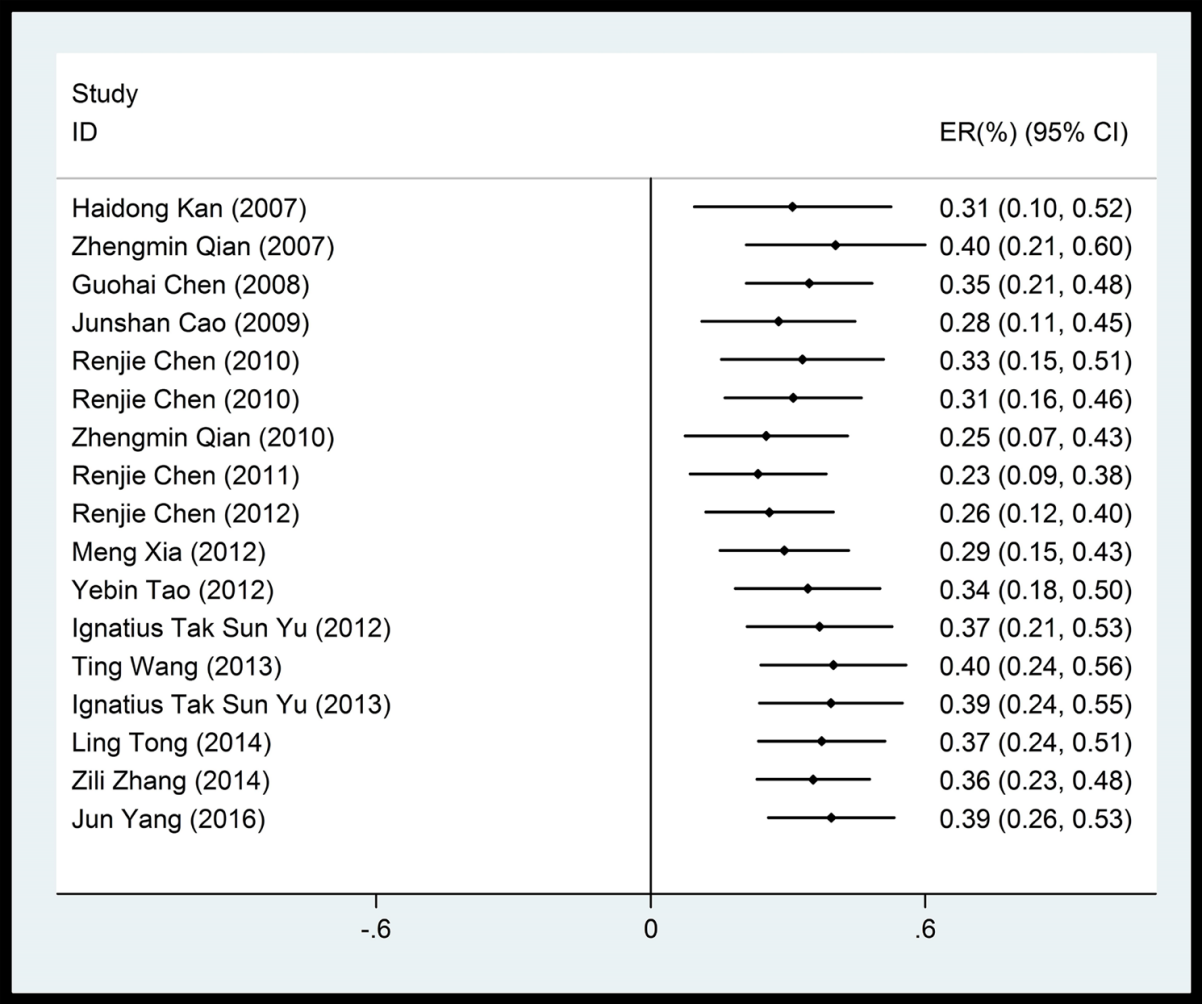
Accumulative meta-analysis of the association between PM_10_ and cardiovascular mortality

PM_2.5_ was also significantly associated with a mean of 0.68% (95% CI: 0.39%–0.97%) increase in cardiovascular mortality per 10 μg/m^3^ increase in concentration, despite significant heterogeneity among studies (90.4%, Figure 4). No time-dependent effect was observed in the adverse impact of PM_2.5_ on cardiovascular mortality (Figure 5).

### Gaseous air pollutants and cardiovascular mortality

Shot-term exposure to gaseous pollutants yielded robust association with cardiovascular mortality. (Table 2) For each 10 μg/m^3^ increase in O_3_ and SO_2_ concentration, pooled ERs of cardiovascular mortality was 0.62% (95% CI: 0.33%–0.92%) and 0.75% (95% CI: 0.42%–1.09%), respectively. Notably, NO_2_ conferred a 1.12% (95% CI: 0.76%–1.48%) increase in cardiovascular mortality per 10 μg/m^3^ increase in concentration. The heterogeneity was significant among studies reporting these gaseous pollutants (NO_2_, 66.9%; SO_2_, 74.2%; O_3_, 61.5%). For forest plots see details in Online Supplement.

**Table 2:** Pooled outcomes of the cardiovascular health effect of gaseous pollutants

	NO_2_	SO_2_	O_3_
**Number of estimates**	12	9	6
**Model**	Random-effect	Random-effect	Random-effect
**Heterogeneity I^2^ (%)**	66.9	74.2	61.5
**Summary ER (%) (95% CI)**	1.12 [0.76, 1.48]	0.75 [0.42, 1.09]	0.62 [0.33, 0.92]

ER, Excess risk (ER) percentages and 95% confidence intervals for different causes of mortality. CI, confidence interval. PM, Particulate matter.

### Subgroup analysis

Subgroup analyses were conducted by stratifying the age, gender, the different lag patterns, temperature, and concentrations of air pollutants (Table 3). People aged greater than 65 years were more susceptible to the adverse impacts conferred by air pollution. Males appeared to be less susceptible to the adverse effects associated with air pollution. The more robust associations were seen at lag 1 day for all pollutants except for PM_2.5_. Among all pollutants, greater cardiovascular mortality was observed in lower temperature. In most of the sub-group analysis, the heterogeneity remained significant, except for the warm seasons.

**Table 3:** Subgroup-analysis of each pollutant and cardiovascular mortality

Pollutant	Outcome	Number of estimates	Heterogeneity I^2^ (%)	Statistics Model	Summary ER (%) (95% CI)
PM_2.5_					
	Lag 0	4	49	Random-effect	0.40 [0.23, 0.58]
	Lag 1	3	90	Random-effect	0.14 [−0.23, 0.52]
	Lag 2	2	77	Random-effect	−0.01 [−0.22, 0.01]
	Lag 0–1	2	71	Fixed-effect	0.77 [0.42, 1.12]
	Warm	2	19	Fixed-effect	0.67 [0.21, 1.14]
	Cool	2	75	Random-effect	0.68 [−0.35, 1.71]
PM_10_					
	Lag 0	9	76	Random-effect	0.27 [0.10, 0.44]
	Lag 1	6	80	Random-effect	0.35 [0.12, 0.59]
	Lag 2	4	63	Random-effect	0.03 [−0.21, 0.27]
	Lag 0–1	12	84	Random-effect	0.47 [0.30, 0.64]
	Male	3	73	Random-effect	0.30 [0.06, 0.55]
	Female	3	80	Random-effect	0.56 [0.14, 0.97]
	Warm seasons	4	73	Random-effect	0.44 [−0.07, 0.96]
	Cool seasons	4	80	Random-effect	0.46 [0.18, 0.73]
	Age > 65 years	5	78	Random-effect	0.50 [0.23, 0.76]
	Age ≤ 65 years	5	80	Random-effect	0.33 [0.01, 0.65]
NO_2_					
	Lag 0	3	75	Random-effect	0.44 [−0.46, 1.34]
	Lag 1	2	77	Random-effect	1.11 [0.12, 2.11]
	Lag 0–1	8	86	Random-effect	1.33 [0.73, 1.93]
	Male	3	86	Random-effect	0.80 [0.08, 1.51]
	Female	3	64	Random-effect	1.08 [0.48, 1.69]
	Warm seasons	3	0	Fixed-effect	0.13 [−0.25, 0.50]
	Cool seasons	3	90	Random-effect	1.96 [0.33, 3.60]
	Age > 65 years	4	86	Random-effect	1.27 [0.55, 1.99]
	Age ≤ 65 years	4	50	Fixed-effect	0.40 [0.20, 0.61]
SO_2_					
	Lag 0	3	87	Random-effect	0.38 [−0.45, 1.20]
	Lag 1	4	87	Random-effect	0.72 [0.05, 1.39]
	Lag 2	2	41	Fixed-effect	0.12 [−0.13, 0.38]
	Lag 0–1	8	82	Random-effect	0.61 [0.23, 1.00]
	Male	2	59	Random-effect	0.56 [0.08, 1.04]
	Female	2	87	Random-effect	0.99 [−0.10, 2.09]
	Warm seasons	3	3	Fixed-effect	0.21 [−0.01, 0.43]
	Cool seasons	3	86	Random-effect	1.28 [0.39, 2.18]
O_3_					
	Lag 2	2	81	Random-effect	0.40 [−0.04, 0.84]
	Lag 0–1	2	76	Random-effect	1.51 [−1.32, 4.33]
	Warm seasons	3	36	Fixed-effect	0.43 [0.15, 0.71]
	Cool seasons	4	91	Random-effect	1.72 [−0.71, 4.15]

ER, Excess risk (ER) percentages and 95% confidence intervals for different lag times of mortality, different age groups, different genders and different temperature for each increment of 10 μg/m3 in pollutant concentrations. CI, confidence interval. PM, Particulate matter

### Publication bias

Funnel plots suggested symmetric distribution of studies (see Online Supplement). Begg’s and Egger’s test indicated insignificant publication bias for studies included in our analysis (Table 4).

**Table 4:** Assessment for publication bias stratified by gaseous and particulate air pollutants

	PM_2.5_	PM_10_	NO_2_	SO_2_	O_3_
**Number of estimates**	10	17	12	9	6
***P* value for Begg’s test**	0.049	0.058	0.631	0.251	0.060
***P* value for Egger’s test**	0.124	0.758	0.020	0.644	0.048

ER, Excess risk. CI, confidence interval. PM, Particulate matter.

## DISCUSSION

Cardiovascular diseases include disorders of the heart (arrhythmia, coronary vessel and vascular disease, heart failure) and blood vessels (peripheral arterial diseases and venous thrombosis), particularly the diseases related to vessels supplying blood to the brain (ischemic and hemorrhagic stroke). Taken together, these disorders constitute the leading cause of death across the globe, with low- and middle-income countries being most significantly affected. A substantial number of environmental factors have been found to exert a critical adverse impact on the risk, progression, and severity of cardiovascular diseases. There are growing concerns of cardiovascular mortality related to air pollution, although it remains poorly characterized in China. In this meta-analysis, we have pooled 30 epidemiological studies which focused on the association between short-term exposure to pollution and cardiovascular mortality, demonstrating positive correlations between air pollutant levels and cardiovascular mortality. Specifically, we observed 0.39% and 0.68% higher ERs for total cardiovascular mortality, per 10 μg/m^3^ increases in PM_10_ and PM_2.5_, respectively. Meanwhile, each 10 μg/m^3^ increase in NO_2_, SO_2_ and O_3_ was associated with 1.12%, 0.75% and 0.62% greater ERs in cardiovascular mortality. Similar outcomes were reported in a previous meta-analysis from China, in which each 10 μg/m^3^ increase in PM_10_, PM_2.5,_ SO_2_, NO_2_, and O_3_ concentrations corresponded to an increase in cardiovascular mortality by 0.43%, 0.44%, 0.85%, 1.46%, and 0.45%, respectively [21]. Another study has documented the increase of 0.36% and 0.63% per 10 μg/m^3^ increase in PM_10_ and PM_2.5_, which was also comparable to our findings [22].

Admittedly, pooled estimates of PM_10_ derived from developed countries were greater than that from China. Each 10 μg/m^3^ increase in PM_10_ was associated with 0.53% and 0.68% greater cardiovascular mortality in Europe [17] and USA [23], respectively. But our pooled estimates for PM_2.5_ of 0.68% appeared lower than those reported in developed countries. Per 10 μg/m^3^ increase in PM_2.5_, the pooled ERs were 0.85% for cardiovascular mortality in a multicity time-series analysis from 112 US cities [24]. In another time-series study from 27 US cities, each 10 μg/m^3^ increase in PM_2.5_ correlated with a pooled ER of 0.94% in cardiovascular mortality [18]. Because of significantly higher average of pollutants concentrations in Chinese cities, the exposure-response coefficient derived from western cities cannot be directly extrapolated to China.

We noted greater adverse effects of PM_2.5_ on cardiovascular mortality than those of PM_10_, which has been supported by earlier epidemiological studies [25, 26]. A potential interpretation is that PM_10_ deposited preferentially in larger airways, whereas PM_2.5_ or ultrafine particulate matter could penetrate to more distal airways or even the alveoli [27, 28]. It has been proven that PM_2.5_ enters cardiovascular system via inhalation into the lungs, which promotes local inflammatory response that “spills over” into the circulation, where soluble and cellular mediators may promote systemic oxidative stress and inflammation that affect the heart and vessels [29]. This systemic effect can also be amplified by effects on adipose and liver tissue, promoting the release of adipokines and acute-phase reactants, which can alter vascular tone, resulting in insulin resistance, dyslipidemia and hypercoagulability [30]. Therefore, greater emphasis should be placed on the adverse effects conferred by PM_2.5_.

Temperature is usually considered as a confounder of air pollution [31], and its impacts on cardiovascular mortality associated with air pollution remain controversial [32]. Consistent with previous studies [33, 34], we noted that low atmospheric temperature was associated with greater cardiovascular mortality, although confounding by other meteorological factors cannot be excluded. The altered cardiovascular biomarker profiles in healthy adults associated with ambient temperature changes may help explain the temperature-related cardiovascular mortality. A 10°C decrease at 2-d average daily temperature were associated with mean increases of 2.5%, 1.6%, 2.7%, 5.5% and 2.0% in biomarker levels for systemic inflammation, coagulation, systemic oxidative stress, antioxidant activity and endothelial function, respectively [35]. We also observed greater adverse cardiovascular mortality in females than in males, although previous studies yielded conflicting results [36]. These findings indicated that effect-modifiers should be taken into account when interpreting the impact of air pollutants on cardiovascular mortality.

Except for metropolitans, the adverse effects conferred by air pollutants remain significant in other cities of northwest China. More stringent regulations on improving air quality, coupled with implementation of routine atmosphere monitoring, are urgently needed. In light of different impacts on cardiovascular mortality, we propose nation-wide dynamic monitoring of pollutant levels, which will better inform citizens at risk of developing cardiovascular events in case of abrupt increase in pollutant levels.

Limitations of our study include the significant heterogeneity among all pollutants except for PM_2.5_ and O_3_. Factors that could potentially explain for the significant heterogeneity may include different study designs and methods for statistical analysis, the diverse population characteristics, diverse regions where study participants are residing, and the methods for measurement and recording, etc. However, pooled estimates showed consistently remarkable adverse effect among all pollutants, and the effect size was unchanged in subgroup analyses. We only calculated pooled estimates based on single-pollutant model without adjustment. The effects of air pollution on cardiovascular mortality might have been overestimated because our pooled estimates were merely based on short-term exposures to air pollution. Our study estimated the health effect on more general population; however, the effects were likely to be greater in patients with pre-existing cardiovascular diseases. Finally, most cities in our study were mega-cities that harbored dense population, therefore it remains unclear whether our findings could be extrapolated to cities with smaller scales.

In summary, short-term exposure of air pollution is associated with greater cardiovascular mortality. Our results reinforce the public health importance of surveillance of air pollution, which would help inform policy-makers to establish more stringent regulations that would mitigate air pollutant levels. Despite small pooled estimates, the impact is substantial for the entire population. More high-quality studies in China are urgently needed to identify the exposure-response effect in order to determine the susceptible population, making relevant policy for further prevention of the detrimental effects due to air pollution.

## MATERIALS AND METHODS

### Literature search and selection

Systematic literature search was performed for epidemiological studies conducted in China that examined cardiovascular disease mortality and hospital admissions in association with short-term exposure to air pollutants, including particulate matter (PM_10_, PM_2.5_) and gaseous pollutants (NO_2_, SO_2_, O_3_).

We searched PubMed, Web of Science, Cochrane library, Wan Fang, and CNKI databases for literature published in English or Chinese, up to January 2017, using the terms: (1) PM_2.5_, PM_10_, NO_2_, SO_2_, O_3_, air pollutants, air pollution; (2) Cardiovascular diseases, adverse effect, mortality, death; (3) time-series, case-crossover, cohort studies; (4) China, Chinese. Additional literature was manually retrieved to identify studies not included during the initial search. Selection of studies was based on viewing the titles, abstracts and full-length articles.

We focused on time-series or case-crossover studies that reported excess risk (ER) and the 95% confidence intervals (95% CI) of cardiovascular mortality in association with short-term exposure to air pollutants (NO_2_, SO_2_, O_3_, PM_2.5_, and PM_10_) in China. Only single-pollutant model results and studies on human were included. Following inclusion criteria were used: (1) All epidemiological studies, involved the health impact of exposure to mentioned pollutants in the Chinese population; (2) original studies expressed quantitative exposure–response relationships between mentioned pollutants and cardiovascular health outcomes (excess risk[ER], and their 95%confidence intervals [95% CI]); (3) subjects were not from specific high-risk groups (e.g. smokers or children); (4) the health outcomes were cardiovascular mortality.

### Data extraction and meta-analysis

Data were extracted independently by two investigators (H.R.L and F.Y.C.) and discrepancy was adjudicated by a third investigator (L.Z.). For selected studies, the title, authors, location, year of publication, study period, study design, number of events, type of pollutants and health outcomes were extracted and entered into an electronic database.

Association between short-term exposure of air pollution and cardiovascular mortality was frequently determined for the duration of several days prior to the events, showing the estimates as single time lags (e.g. lag 1) or cumulative lags (e.g. lag 0–1 or 0–2). For eligible studies, we selected the lags to pooled risk estimates with the following rules: 1) the estimate could be directly included if only one lag estimate was presented; 2) if multiple lags were presented, we sequentially selected with the following criteria: [37, 38] (a) the *a-priori* lag; (2) the lag with the greatest statistical significance; (3) the lag with the largest effect estimate; (4) the lag with the shortest period.

We did subgroup analyses according to different lag patterns (0, 1, 2, 0–1, 0–2 days), age (≤ 65 vs. > 65 years), gender (male vs. female), and temperature (warm vs. cold).

Excess risks (ERs) were pooled for standardized estimates with incremental concentration of 10 μg/m^3^ [22]. We used Cochran’s *X*^2^ test and I^2^ to examine the heterogeneity among effect estimates [39]. *P* > 0.05 indicated homogeneity, otherwise heterogeneity among estimates. I^2^ statistics of 0∼25, 25∼50 and > 50 indicated low, moderate, and significant heterogeneity, respectively [40]. We chose I^2^ > 50 as an indicator of significant heterogeneity. Random-effect model was applied to pool estimates in case of significant heterogeneity; otherwise, fixed-effect model was applied [39]. Study bias was detected using funnel plot, based on Begg’s and Egger’s test [41, 42]. Statistical significance was taken for *P* < 0.05. All analyses were conducted with STATA version 12.0 (Stata Corporation College Station, TX, USA).

## SUPPLEMENTARY MATERIALS FIGURES AND TABLE




