# Supplementary material for: Association between air pollution and cardiovascular mortality in China: a systematic review and meta-analysis
Source: Oncotarget. 2017 Aug 9;8(39):66438–48. doi: 10.18632/oncotarget.20090 (PMC5630425; doi:10.18632/oncotarget.20090)
Supplement: Supplementary file 1 [file oncotarget-08-66438-s001.pdf]

Association between air pollution and cardiovascular mortality in China: a systematic review and meta-analysis

SUPPLEMENTARY MATERIALS

Supplementary Table 1: Population and concentration. See Supplementary_Table_1

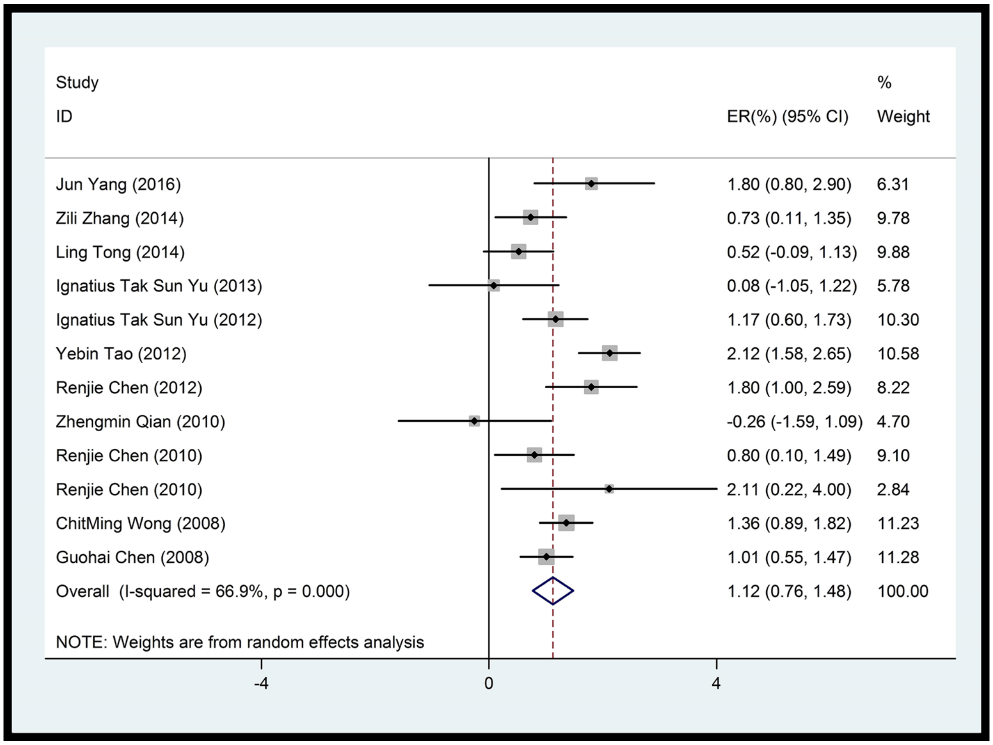

Supplementary Figure 1: Forest plot of the association between NO2 and cardiovascular mortality.

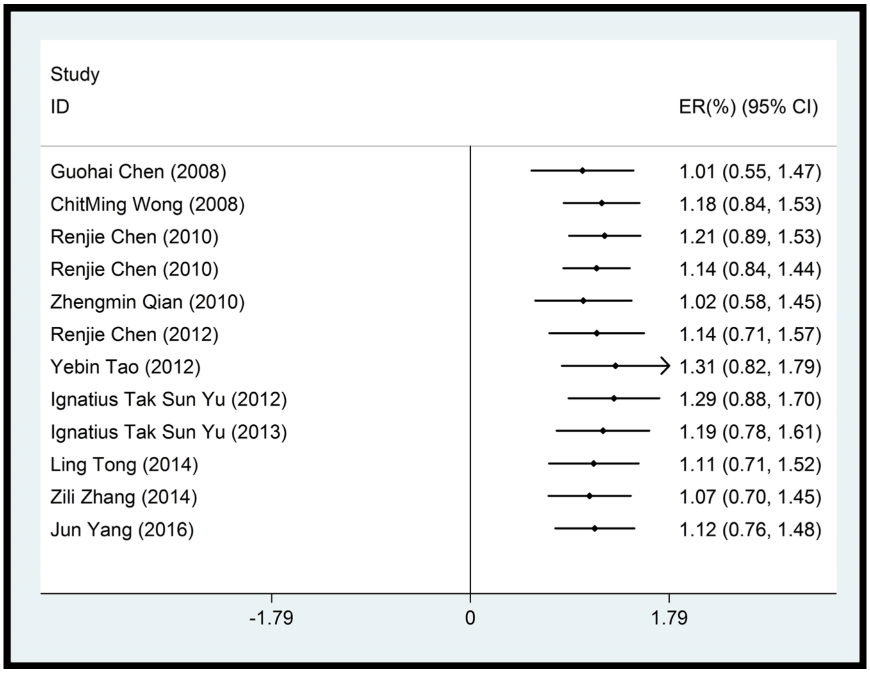

Supplementary Figure 2: Accumulative meta-analysis of the association between NO2 and cardiovascular mortality.

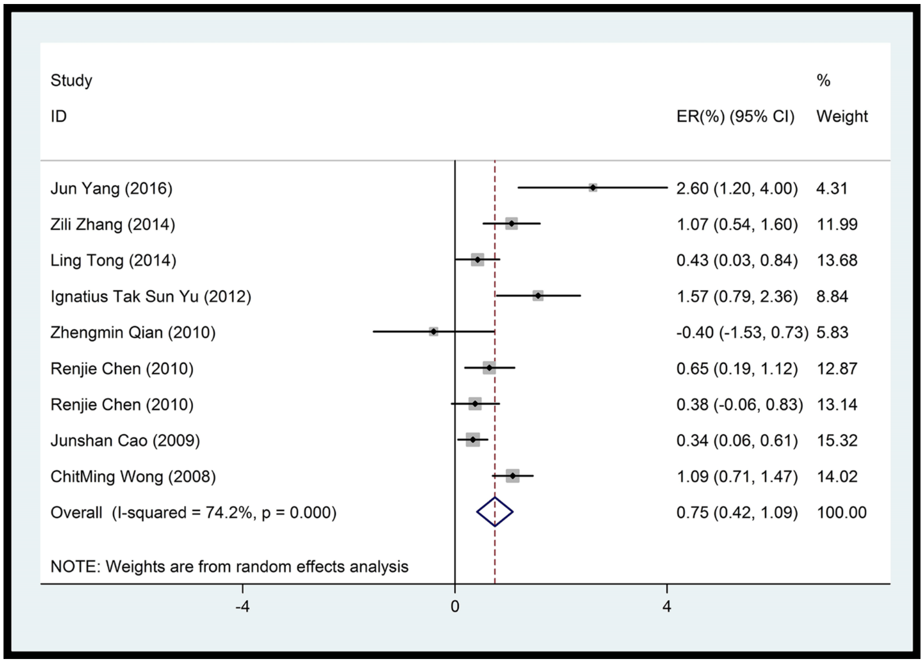

Supplementary Figure 3: Forest plot of the association between SO2 and cardiovascular mortality.

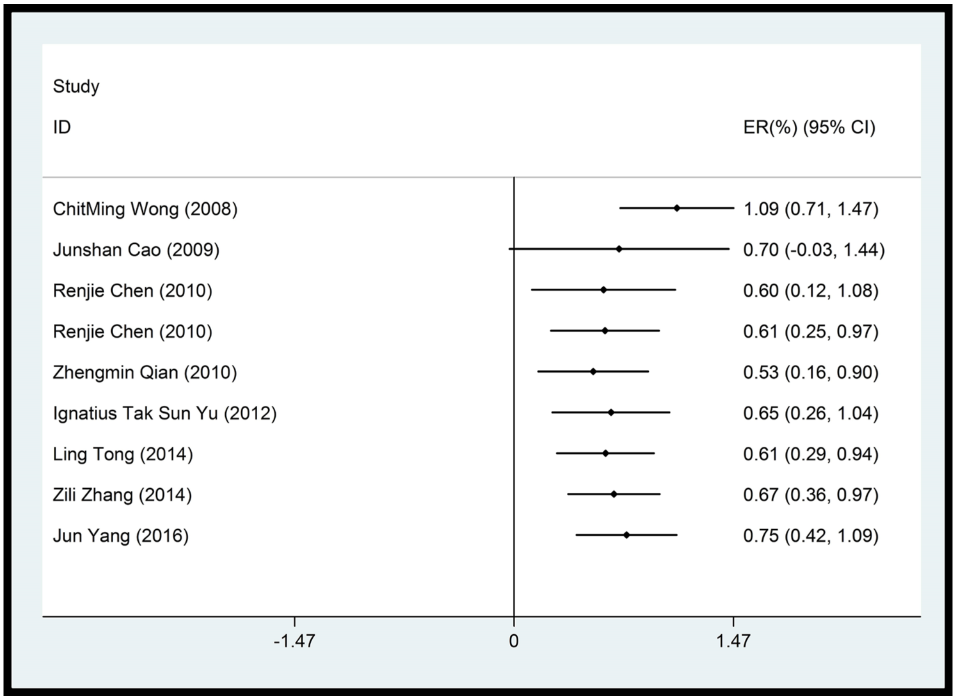

Supplementary Figure 4: Accumulative meta-analysis of the association between SO2 and cardiovascular mortality.

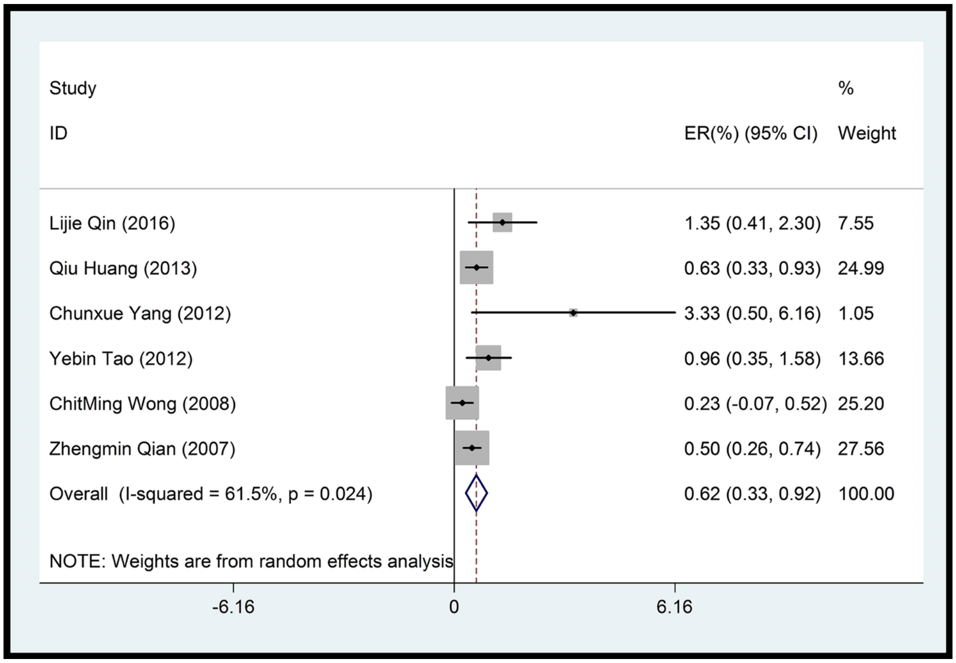

Supplementary Figure 5: Forest plot of the association between O3 and cardiovascular mortality.

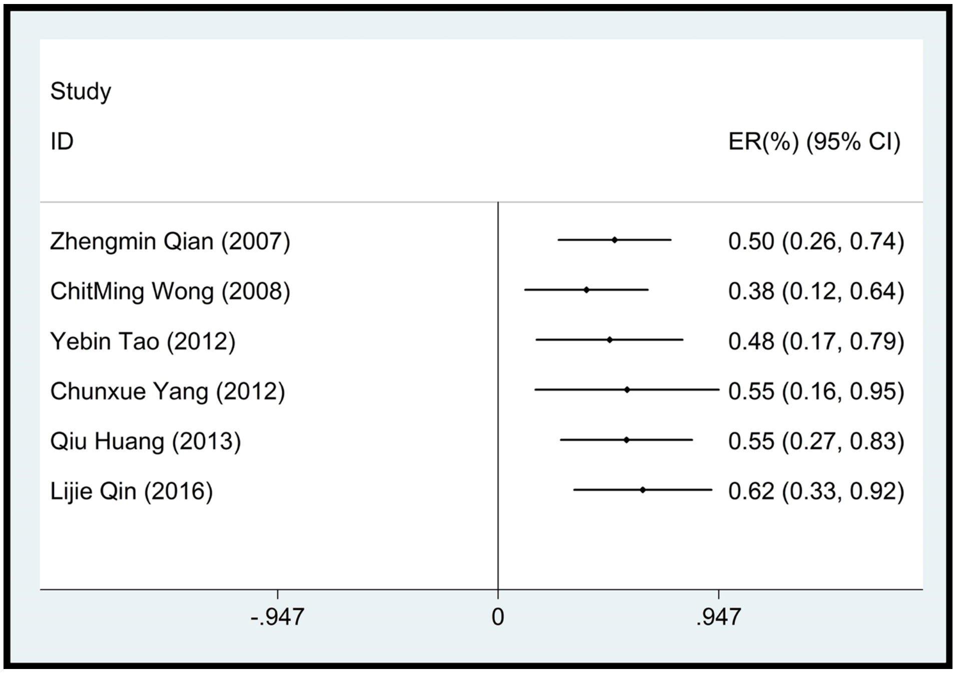

Supplementary Figure 6: Accumulative meta-analysis of the association between O3 and cardiovascular mortality.

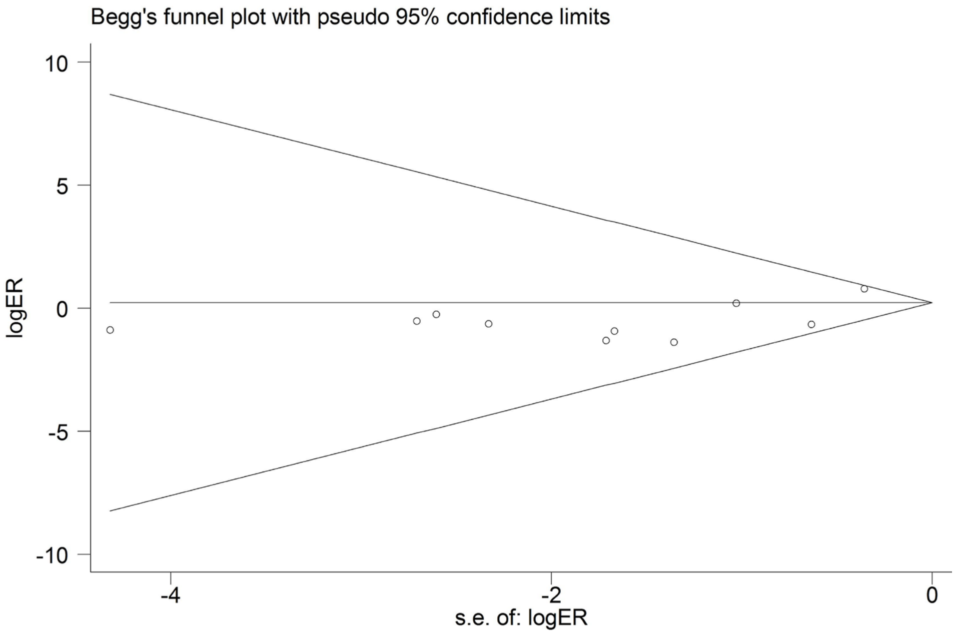

Supplementary Figure 7: Publication bias of the association between PM2.5 and cardiovascular mortality.

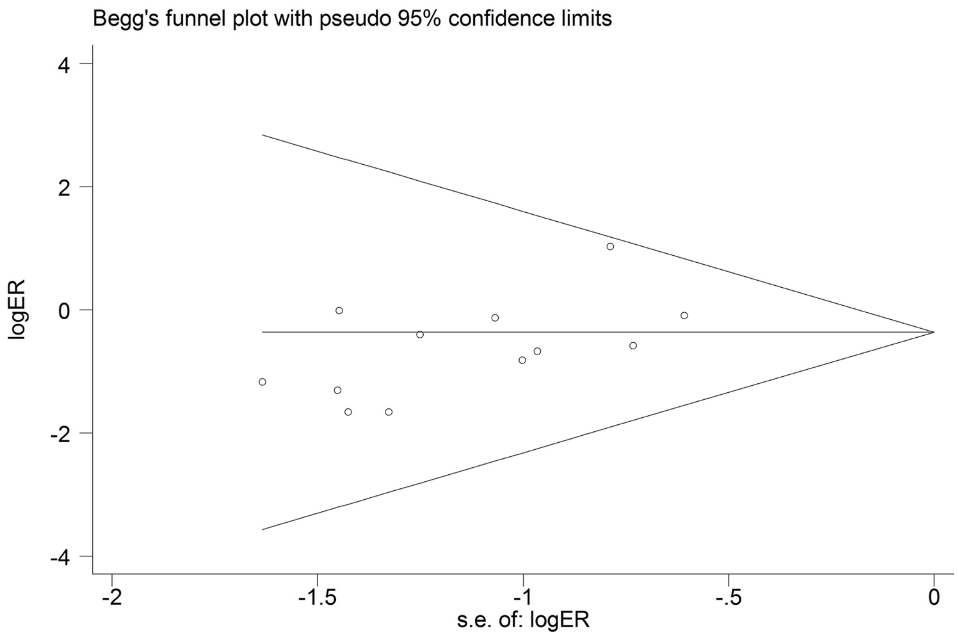

Supplementary Figure 8: Publication bias of the association between PM10 and cardiovascular mortality.

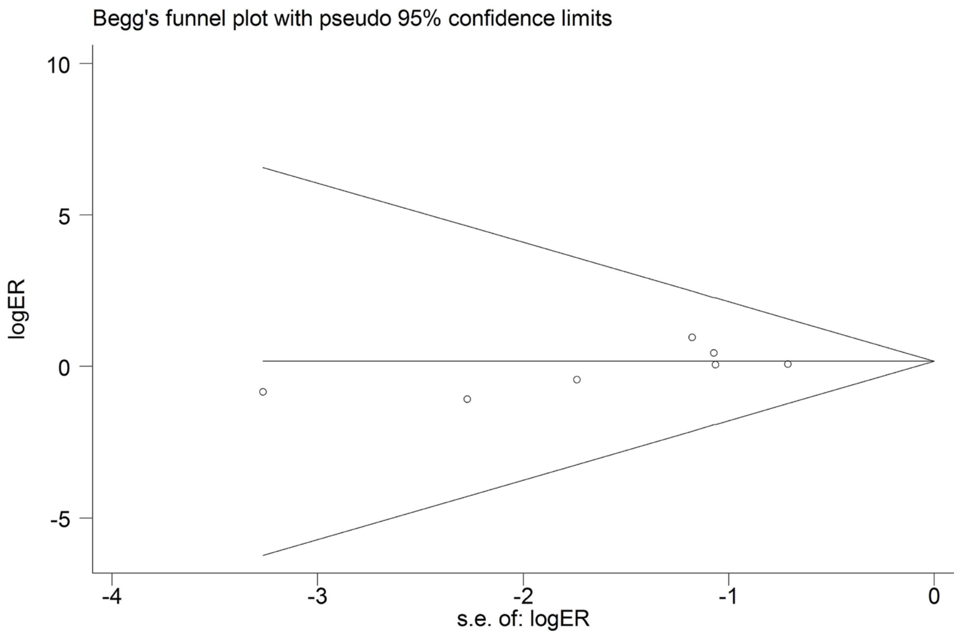

Supplementary Figure 9: Publication bias of the association between NO2 and cardiovascular mortality.

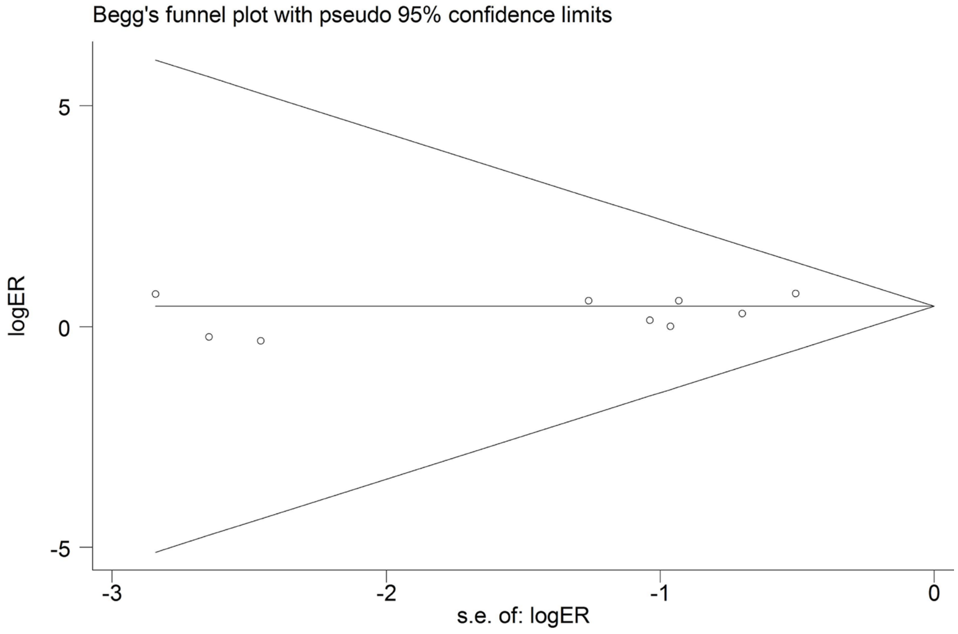

Supplementary Figure 10: Publication bias of the association between SO2 and cardiovascular mortality.

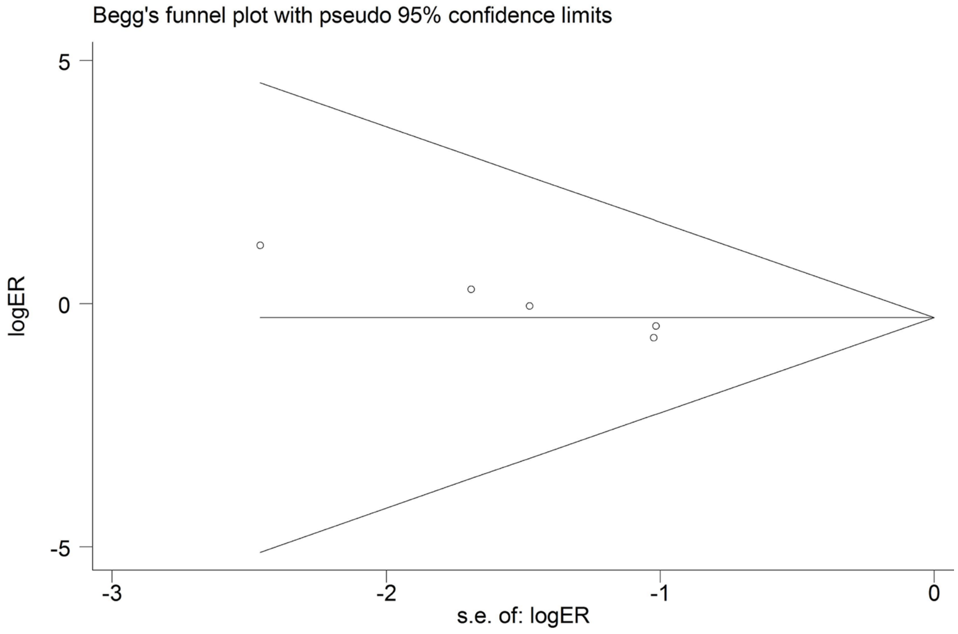

Supplementary Figure 11: Publication bias of the association between O3 and cardiovascular mortality.
