# Supplementary material for: Association between air pollution and cardiovascular mortality in China: a systematic review and meta-analysis
Source: Oncotarget. 2017 Aug 9;8(39):66438–48. doi: 10.18632/oncotarget.20090 (PMC5630425; doi:10.18632/oncotarget.20090)
Supplement: Supplementary file 2 [file oncotarget-08-66438-s002.docx]

Supplementary Table 1: Population and concentration

|  | | | | | | |
| --- | --- | --- | --- | --- | --- | --- |
| title | population | NO_2_(μg/m^3^) | SO_2_(μg/m^3^) | PM_10_(μg/m^3^) | PM_2.5_(μg/m^3^) | O_3_(μg/m^3^) |
| Impact of haze and air pollution-related hazards on hospital admissions in Guangzhou, China | 1,461 | 56 | 32 | 62 |  |  |
| Effect of ambient air pollution on daily mortality rates in Guangzhou, China | NG | 47.69 | 43.3 | 59.49 |  |  |
| Synergy between particles and nitrogen dioxide on emergency hospital admissions for cardiac diseases in Hong Kong | 3,652 | 58 |  | 52.8 |  |  |
| Estimating years of life lost from cardiovascular mortality related to air pollution in Guangzhou, China | NG | 72.3 | 59 | 85.3 |  |  |
| Alternative ozone metrics and daily mortality in Suzhou: the China Air Pollution and Health Effects Study (CAPES) | 37,571 |  |  | 89.4 |  | 35.7 |
| A time-stratified case-crossover study of fine particulate matter air pollution and mortality in Guangzhou, China | 58,400 | 65.8 | 50 |  | 70.1 |  |
| Relationship between fine particulate air pollution and ischaemic heart disease morbidity and mortality | 369,469 |  |  |  | 96.2 |  |
| Public Health and Air Pollution in Asia (PAPA): a multicity study of short-term effects of air pollution on mortality | NG | 44.7 | 13.2 | 52 | . | 59.4 |
| Association between ambient particulate matter and daily cause-specific mortality in Tanggu, Tianjin Binhai New Area, China | 13,273 | 44 | 60.2 | 99.2 |  |  |
| Promoted relationship of cardiovascular morbidity with air pollutants in a typical Chinese urban area | NG | 13.5 | 75.6 | 78.9 |  |  |
| Estimated acute effects of ambient ozone and nitrogen dioxide on mortality in the Pearl River Delta of southern China | NG | 53.9 | 55.2 | 81 |  | 78.2 |
| Cool and dry weather enhances the effects of air pollution on emergency IHD hospital admissions | 3,652 | 58 | 19.5 | 52.8 |  | 39.8 |
| Seasonal association between ambient ozone and mortality in Zhengzhou, China | 70,443 | 43.52 | 28.18 |  | 74.23 |  |
| Seasonal Pattern of the Acute Mortality Effects of Air Pollution | NG | 49.5 | 34.5 | 143 |  |  |
| Association of daily cause-specific mortality with ambient particle air pollution in Wuhan, China | 89,131 | NG | NG | NG | NG | NG |
| Temperature modifies the acute effect of particulate air pollution on mortality in eight Chinese cities | NG |  |  | 65 |  |  |
| Fine particulate air pollution and daily mortality in Shenyang, China | 60,938 | 37 | 55 |  | 75 |  |
| Mortality burden of ambient fine particulate air pollution in six Chinese cities: Results from the Pearl River Delta study | 316,305 | 47.41 | 19.51 |  | 47.89 | 56.34 |
| Ambient temperature enhanced acute cardiovascular-respiratory mortality effects of PM2.5 in Beijing, China | 22,489 |  |  |  | 82.02 |  |
| Differentiating the effects of fine and coarse particles on daily mortality in Shanghai, China | 79,530 | NG | NG | NG | NG | NG |
| Visibility, air quality and daily mortality in Shanghai, China | 668 | 61.8 | 57.7 | 107.9 | 56.4 | 77 |
| Seasonal variation of chemical species associated with short-term mortality effects of PM(2.5) in Xi'an, a Central City in China | NG | 16.2 | 38.1 |  | 176.7 |  |
| Differentiating the associations of black carbon and fine particle with daily mortality in a Chinese city | 77,964 |  |  |  | 53.9 |  |
| Association of particulate air pollution with daily mortality: the China Air Pollution and Health Effects Study | NG | NG | NG | NG | NG | NG |
| Ambient air pollution and daily mortality in Anshan, China: a time-stratified case-crossover analysis | NG | 25.5 | 59 | 110.9 |  |  |
| Coarse particles and mortality in three Chinese cities: the China Air Pollutio{Chen, 2011 #889}n and Health Effects Study (CAPES) | NG |  |  | 172 | 82 |  |
| Association of particulate air pollution with daily mortality: the China Air Pollution and Health Effects Study | NG |  |  | 111 |  |  |
| Ambient air pollution and hospital admission in Shanghai, China | NG | 57 | 56 | 87 |  |  |
| Short-term effects of ambient gaseous pollutants and particulate matter on daily mortality in Shanghai, China | 173,911 | 66.6 | 44.7 | 102 |  |  |
| Association of ambient air pollution with hospital outpatient and emergency room visits in Shanghai, China | 7,831,947 | 57 | 56 | 87 |  |  |
